# Reference intervals for reproductive hormones in Chinese children aged 0–14 years based on the PRINCE study

**DOI:** 10.1080/07853890.2026.2650008

**Published:** 2026-04-10

**Authors:** Ying Liu, Xiaoyi Tian, Wenqi Song, Xin Ni

**Affiliations:** ^a^ Department of Clinical Laboratory Center, Beijing Children’s Hospital, Capital Medical University, National Center for Children’s Health, Beijing, P.R. China; ^b^ Department of Otolaryngology, Head and Neck Surgery, Beijing Children’s Hospital, Capital Medical University, National Center for Children’s Health, Beijing, P.R. China

**Keywords:** Reference intervals, sex hormones, reproductive hormones, Chinese children, PRINCE

## Abstract

**Background:**

The concentrations of reproductive hormones considerably change during growth and development. Establishing accurate age- and sex-specific reference intervals for these hormones is important for monitoring development during childhood. Therefore, this study used data from the Paediatric Reference Intervals in China (PRINCE) study to establish and validate reference intervals (RIs) for sex and reproductive hormones in Chinese children.

**Materials and methods:**

This study is based on healthy children and adolescents from the PRINCE cohort. Serum samples were collected to determine age- and sex-specific RIs for luteinizing hormone, follicle-stimulating hormone, testosterone, oestradiol, progesterone and prolactin using a non-parametric method recommended by the Clinical and Laboratory Standards Institute guidelines.

**Results:**

The study included 1,707 healthy children (819 males and 888 females). Paediatric RIs for six reproductive hormones were established and stratified into distinct age and sex partitions. Key hormonal surges marked pubertal transition: in females, the upper limit of oestradiol more than doubled at age 9 years, while follicle-stimulating hormone began rising at age 8 years. In males, the upper limit of testosterone increased several-fold at age 11 years. Luteinizing hormone rose from age 9 in both sexes. Prolactin remained stable from ages 1–14, while progesterone increased in females after age 11. The RIs were successfully validated in 280 samples.

**Conclusion:**

This study is the first to establish and validate RIs for sex and reproductive hormones in Chinese children aged 0–14 years, thereby providing evidence-based support for interpreting laboratory test results and establishing clinical standards for paediatric endocrine disorders.

## Introduction

In paediatric populations, the diagnosis, monitoring and treatment of endocrine and fertility-related diseases, as well as the assessment of sexual development, often rely on the measurement of reproductive hormone concentrations. To aid clinical decision-making and improve diagnostic accuracy, laboratory test results are interpreted based on reference intervals (RIs), also known as reference standards or “normal ranges” [1]. RIs are values that encompass a specific percentage (typically 95%) of a defined population, which is highly useful for diagnosing, prognosing and monitoring the treatment of diseases, as well as performing overall health assessments [2]. Selecting representative reference populations and analytical methods is crucial for determining appropriate RIs, which have been clearly defined by the Clinical and Laboratory Standards Institute (CLSI) in the EP28-A3c document [3].

Accurate diagnosis and management of paediatric endocrine diseases depend on RIs for reproductive hormone, which are essential for pubertal development. Hormone change continuously throughout childhood. For example, the concentrations of gonadal hormones, such as oestradiol (E2), testosterone (T) and progesterone (PRGE), exhibit complex change patterns [4] that differ significantly from those in adults [5,6]. Therefore, the assessment of hormone should rely on RIs specific to local children and ideally customized for different age and sex groups.

Globally, an increasing number of studies have emphasized the need for paediatric RIs [1,7–11]. In 2008, the CLSI published the “Guidelines for Defining, Establishing and Verifying Reference Intervals” [3], which recommend recruiting at least 120 healthy reference individuals per partition to establish RIs [3]. While this goal is relatively feasible in adult populations, the dynamic physiology of children and adolescents often requires multiple age- and sex-specific partitions, which significantly increases the sample size requirements.

The Paediatric Reference Intervals in China (PRINCE) project aimed to generate RIs for commonly used laboratory measurements in clinical practice to achieve more precise disease management. Previous studies have primarily focused on biochemical and hematological analytes, and RIs have been widely applied in various fields [12,13]. This study aimed to build on the progress made by the PRINCE project and establish sex and reproductive hormone RIs for pubertal development in Chinese children.

## Methods

### Study design and sample collection

This cross-sectional study utilized data from healthy children aged 0–14 years enrolled in the PRINCE study during the period from January 2017 to August 2018. The cohort included infants under 6 months of age; however, the number of participants in this narrow neonatal/infant window was limited. Participants with acute or chronic diseases and those with a recent condition (<2 weeks before testing) that could affect the laboratory results were excluded. Aligning with the WS/T780 industry standard, data cleaning focused on age-specific criteria. Accordingly, participants whose body mass index fell outside the normal range for their age and sex—defined as a BMI-for-age z-score less than −2 SD (underweight) or greater than +2 SD (overweight/obese) according to the WHO child growth standards—were excluded [14,15]. Participants’ pubertal development was assessed using a web-based tool developed using the standardized Marshall and Tanner criteria [16,17]. The tool facilitated self-assessment of breast (B1-B5) and pubic hair (PH1-PH5) stages in girls, and genital (G1-G5) and pubic hair (PH1-PH5) stages in boys. All assessments were conducted online through the PRINCE data collection platform (access: http://1.202.139.123/). Based on contemporary consensus for Chinese children, individuals were excluded from the reference population if they exhibited signs of precocious puberty (onset of breast development B2 before age 8 in girls, or testicular volume >4 mL or genital stage G2 before age 9 in boys) or delayed puberty (absence of breast development by age 13 in girls, or absence of testicular enlargement by age 14 in boys).

For post-menarchal female participants, the date of the last menstrual period or the current cycle day was not recorded and samples were not collected at a standardized cycle phase. This approach reflects the common clinical scenario of non-timed sampling. For reference individuals included in the final cohort, Tanner stage data were recorded and categorized (Stage I-V). This comprehensive approach ensured that the data used for further analyses were representative and followed relevant professional standards.

All samples were collected by paediatric nurses who had received specialized training to ensure professionalism and safety. Blood samples were collected regardless of fasting status or, for post-menarchal girls, the phase of the menstrual cycle. This approach was chosen to reflect the real-world clinical scenario where tests are often requested urgently, without regard to cycle timing. Consequently, the established RIs for oestradiol and progesterone in girls of reproductive age represent a single, population-based range that encompasses the physiological variation across the entire menstrual cycle. The serum was separated within 2 h of sample collection. Serum samples were visually inspected, and those with gross haemolysis, icterus, or lipaemia were excluded. Additionally, automated indices (H-index, I-index, L-index) generated by the Atellica^®^ IM analyzer were reviewed, and samples exceeding the manufacturer’s recommended thresholds for interference were also excluded. The serum was aliquoted into cryotubes and stored at −80 °C until analysis; repeated freeze-thaw cycles were prohibited. The samples were tested in a centralized batch within one month of collection.

The Ethics Committee of Beijing Children’s Hospital (No: 2016-53) approved this study. Informed consent was obtained from each participant’s legal representative (parent or guardian) in the case of children aged less than 8 years, Additionally, assent from the children and consent from the legal guardian for children aged 8 years or older were obtained.

### Sample analyses

The serum concentrations of luteinizing hormone (LH), follicle-stimulating hormone (FSH), T, E2, PRGE, and prolactin (PRL) were determined using a chemiluminescent immunoassay (CLIA) on the Atellica^®^ IM analyzer (Siemens Healthineers, Erlangen, Germany). The analytical performance characteristics of the assays, including the limits of blank, limits of detection, limits of quantitation and inter- and intra-assay coefficients of variation (Supplementary Table S1). Quality control was performed per the WS/T402 standard, including regular instrument maintenance and calibration. The laboratory utilized a third-party for internal quality control (Bio-Rad, Hercules, CA, USA). Additionally, we conducted routine checks using standard quality control materials, which were used to daily or regularly (based on the test) monitor the testing precision and enable the timely detection and correction of any potential deviations. The laboratory also actively participated in external quality assessment activities. Although inter-laboratory comparisons were not performed, participation in the external quality assessment activities organized by the National Center for Clinical Laboratories ensured the reliability and accuracy of the testing procedures and allowed for continued improvements in quality. All experimental procedures were performed by specialized technicians trained to adhere to standardized operating protocols.

### Statistical analyses

Hormone concentrations were plotted against age and sex for visual review as per CLSI EP28-A3c. Outlier detection and removal were performed as a pre-specified step prior to RI calculation. We applied Tukey’s method (using 1.5 times the interquartile range as the fence) to identify outliers within each preliminary age-sex partition for all analytes. For analytes with a highly skewed distribution even after age-sex partitioning (specifically E2 and T in pre-pubertal groups), the adjusted Tukey’s method for skewed distributions was employed [18,19]. Identified outliers were reviewed for potential pre-analytical or clinical causes; none were found, and all were excluded from the RI derivation dataset. Age- and sex-specific partitions were identified through a combination of visual and clinical observations and confirmed using statistical methods. Age- and sex-specific partitions were identified through an iterative, multi-step process to ensure they were both physiologically meaningful and statistically justified: (1) data were plotted as scatter or box plots, and trends by age, sex and Tanner stage were reviewed to identify potential inflection points suggestive of physiological transitions; (2) to objectively support the visually identified breakpoints, we employed the Harris & Boyd statistical test for partitioning reference intervals, as adapted for paediatric data and recommended in relevant literature. This test statistically compares distributions between adjacent age groups to determine if separate RIs are warranted and (3) candidate partitions were evaluated to ensure each resulting group had a sufficient sample size (target ≥120, minimum ≥40 for robust method application) to calculate a stable RI. Furthermore, the final age groups were refined to create clinically practical and memorable boundaries where statistically permissible. Age groups were combined into broader partitions when visual inspection and clinical knowledge indicated stable hormone, ensuring sufficient sample size for robust non-parametric estimation. The primary objective was to create partitions that were both physiologically meaningful and statistically viable. For partitions where the sample size met or exceeded 120, non-parametric methods were used to calculate the 2.5th and 97.5th percentiles as the lower and upper limits, along with their 90% confidence intervals (CI). For partitions with a sample size between 40 and 119, robust methods based on Box-Cox transformation were employed as recommended by CLSI EP28-A3c for smaller sample sizes [3]. Partitions with fewer than 40 reference individuals were not established as standalone RIs; instead, these data points contributed to the visual trend analysis and were merged into adjacent, physiologically similar age groups. Statistical analyses were performed using R software (version 4.0.5; R Core Team, Vienna, Austria) and SAS software (version 9.4; Cary, NC, USA).

Following the CLSI EP28-A3c recommendations for reference interval verification (section 5.7.2), a verification by sampling was performed [3]. This standard method is used to assess whether pre-established RIs are applicable to a new setting or subset using a small number of reference samples, as a full re-establishment with 120 samples per partition is often impractical. Twenty healthy reference samples, independent from those used to establish the RIs, were selected for each age-sex partition. Their results were compared against the established RI limits. According to the guideline, if no more than 2 of the 20 results (≤10%, approximating the expected 5% outside the 95% RI) fell outside the RI, the interval was considered verified. If more than 2 results fell outside the RI for a given partition, the verification was considered failed for that partition. In such a case, per CLSI guidance, the RI should be investigated and potentially re-established using a full cohort of ≥120 reference individuals for that specific partition. To explore the physiological relationships between key hormones during development, Spearman rank correlation analyses were conducted between E2 and FSH, E2 and LH, T and FSH, and T and LH. Given the non-linear hormone trajectories, correlations were assessed within two broad developmental windows: the pre-pubertal period (ages 0–8 years) and the peri-/post-pubertal period (ages 9–14 years).

## Results

### Hormone trends and RIs

**Figure 1.**
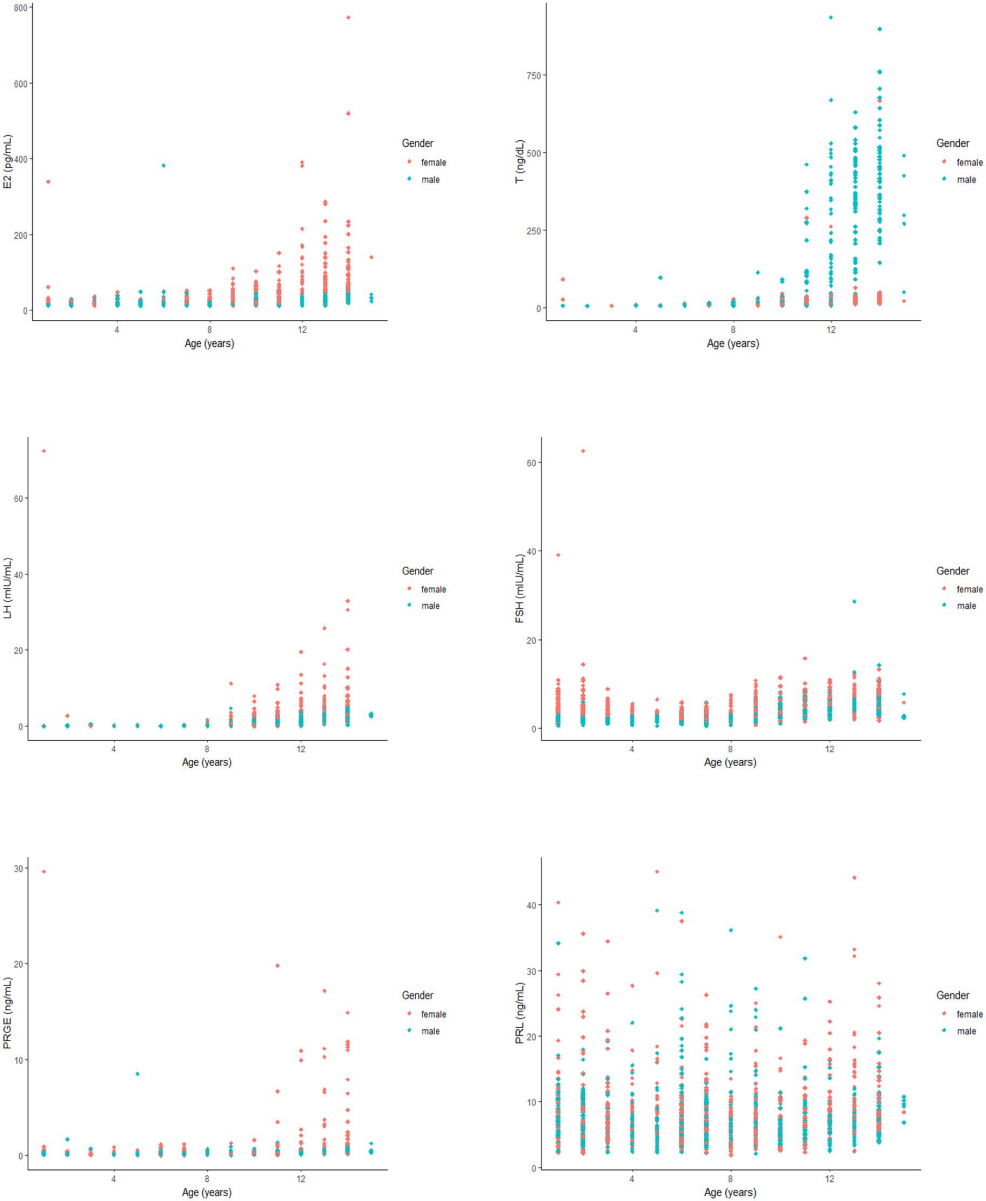
Age- and sex-specific scatterplots for boys (green) and girls (orange), including oestradiol, testosterone, luteinizing hormone, follicle-stimulating hormone, progesterone and prolactin.

The study enrolled 1,717 participants, including 819 males and 888 females. All six hormones intricately and dynamically changed with age (Figure 1). Table 1 presents the numerical age- and sex-based RIs for the six reproductive hormones.

**Table 1.** Reference intervals for reproductive hormones in Chinese children aged 0–14 years old.

Analytes	Female reference interval						Male reference interval					
	Partition	LL	UL	n	90% CI for LL	90% CI for UL	Partition	LL	UL	n	90% CI for LL	90% CI for UL
LH (mIU/mL)	1 to 6 years	0.00	0.12	360	0.00-0.00	0.10-0.17	1 to 3 years	0.00	0.29	171	0.00-0.00	0.20-0.63
	7 to 8 years	0.00	0.23	176	0.00-0.00	0.14-0.60	4 to 8 years	0.00	0.25	331	0.00-0.00	0.20-0.39
	9 years	0.00	4.14	91	0.00-0.00	2.37-7.44	9 years	0.00	2.13	77	0.00-0.00	1.41-3.26
	10 to 12 years	0.00	11.09	131	0.00-0.00	7.89-19.52	10 to 12 years	0.00	3.59	121	0.00-0.02	2.92-5.58
	13 to 14 years	0.73	24.69	126	0.55-0.95	16.34-32.95	13 to 14 years	0.65	5.64	113	0.52-0.82	4.96-6.40
T (ng/dL)	1 to 10 years	<7.00	21.49	679	<7.0-<7.0	19.37-26.68	1 to 10 years	<7.00	18.59	624	<7.00-7.00	15.73- 27.14
	11 to 14 years	9.63	46.55	206	7.18-12.09	41.69-49.78	11 years	<7.00	419.14	40	<7.00-<7.00	274.51-456.03
							12 to 13 years	<7.00	671.08	98	<7.00-23.90	561.04-843.25
							14 years	187.87	857.26	57	153.60-225.95	767.33-957.46
E2 (pg/mL)	1 to 8 years	<11.80	39.53	538	<11.80-<11.80	32.63-47.28	1 to 3 years	<11.80	24.73	171	<11.80-<11.80	20.97-34.67
	9 to 11 years	<11.80	105.48	186	<11.80-<11.80	98.96-151.38	4 to 9 years	<11.80	31.23	409	<11.80-<11.80	28.37-44.75
	12 to 14 years	22.28	286.21	163	19.19-26.23	223.91-519.06	10 to 13 years	<11.80	47.55	182	<11.80-<11.80	42.22-62.25
							14 years	20.69	63.69	57	19.01-22.56	55.66-74.09
FSH (mIU/mL)	1 to 2 years	1.67	11.09	122	0.90-2.83	9.63-14.38	1 to 6 years	0.83	3.12	346	0.62-0.97	2.98-3.62
	3 to 4 years	1.95	6.93	85	1.81-2.10	6.10-7.87	7 to 8 years	0.75	4.22	156	0.38-1.20	3.82-5.82
	5 to 7 years	1.28	4.76	244	0.85-1.43	4.38-5.78	9 to 10 years	1.01	7.03	122	0.99-1.24	6.87-8.13
	8 to 9 years	1.40	8.22	178	0.67-1.68	7.48–10.73	11 years	1.90	8.67	40	1.62-2.20	7.31-10.46
	10 to 11 years	1.91	11.70	94	1.58-2.29	10.65-12.88	12 to 14 years	2.43	10.72	155	1.92-2.61	8.59-28.57
	12 to 14 years	2.13	11.80	163	1.69-2.45	10.85-13.21						
PRL(ng/mL)	1 to 3 years	2.55	29.86	164	2.19-3.25	24.12-40.39	1 to 3 years	2.66	16.90	171	2.32-3.32	12.65-34.19
	4 to 9 years	2.83	20.32	466	2.45-3.08	17.81-25.04	4 to 9 years	2.69	23.58	409	2.41-3.06	19.64-28.28
	10 to 12 years	2.70	21.72	131	2.32-3.03	18.04-35.12	10 to 14 years	2.71	16.33	239	2.46-2.86	15.21-25.76
	13 to 14 years	4.97	31.37	127	2.59-5.56	20.56-44.14						
PRGE (ng/mL)	1 to 10 years	0.05	0.52	679	0.04-0.06	0.48-0.61	1 to 12 years	0.04	0.50	706	0.03-0.05	0.45-0.59
	11 to 12 years	0.10	2.46	78	0.09-0.11	1.28-6.86	13 to 14 years	0.17	0.85	113	0.15-0.19	0.76-0.95
	13 to 14 years	0.16	11.83	126	0.14-0.20	11.00–17.17						

LH: luteinizing hormone; T: testosterone; E2: oestradiol; FSH: follicle-stimulating hormone; PRL: prolactin; PRGE: progesterone.

#### E2

The upper limit of E2 for 9-year-old girls was more than double that for girls under 9 years old, which is a strong indication of the onset of puberty (Figure 1A). Overall, E2 remained relatively stable from birth until the onset of puberty and then increased with age. A small number of isolated, physiologically implausibly high E2 values were observed in pre-pubertal children of both sexes. These values exceeded the upper fence of the adjusted Tukey method by more than 1.5 times the interquartile range. None of these samples showed visual evidence of haemolysis, lipaemia or icterus, and automated interference indices were within acceptable limits. These isolated, physiologically implausible high values are not illustrated in Figure 1 as they were removed prior to final figure generation.

#### T

The upper limit of T for 11-year-old boys was several times higher than that for boys under 11 years old (Figure 1B). T remained relatively stable before puberty and then increased with age. The expected transient rise during ‘mini-puberty’ (approximately 1–6 months of age) is not distinctly visible in the scatter plot. This is likely due to the sparse number of samples in this very narrow age window within our cross-sectional design, coupled with the high individual variability of this transient surge. Consequently, the established RIs for the youngest age group (e.g. 0–1 year) should be interpreted as a general range for early infancy rather than a specific map of the mini-puberty peak.

#### LH

LH maintained a relatively stable level from birth until puberty and then increased with age (Figure 1C). The observed peak LH values in pubertal and adolescent girls, are consistent with the known physiological LH surge during the peri-ovulatory phase of the menstrual cycle. As samples were not timed to cycle phase, these high values represent the upper end of normal physiological variation captured by our ‘all-phase’ reference intervals.

#### FSH

FSH was high in girls before age 2 years and then significantly decreased. This result necessitated different groupings for both sexes (Figure 1D). FSH began to trend upward at age 8 (one year earlier than LH).

#### PRGE

PRGE was relatively stable in boys and young girls. However, the level started to increase at age 11 years in girls and significantly rose at and above age 13 (Figure 1E).

#### PRL

PRL remained stable in both boys and girls from ages 1 to 14 years (Figure 1F).

To provide context for the observed trends in our cohort, smoothed curves representing the central tendency (e.g. median or geometric mean) and the 2.5th/97.5th percentile trajectories for key hormones (LH, FSH, E2, T) in the Chinese children from this study are presented in Supplementary Figure S1.

### Proportion of children with biochemical pubertal activation by age

To address the heterogeneity of pubertal onset within age groups, we analyzed the proportion of individuals exhibiting hormone concentrations above a pre-pubertal threshold within key age groups where the upper reference limit showed a notable surge. For example, within the 9-year-old female group, approximately 22% had an E2 concentration above the pre-pubertal upper limit (39.5 pg/mL), directly illustrating the proportion initiating a biochemical pubertal rise at that age. Similar analyses showed that 29% of 9-year-old girls and 19% of 9-year-old boys had LH above their respective pre-pubertal thresholds (0.23 mIU/mL and 0.25 mIU/mL). For 11-year-old boys, 50% had a T concentration above the pre-pubertal upper limit of 18.59 ng/dL. These results are summarized in Supplementary Table S2.

### Hormone by tanner stage

To illustrate the relationship between the established age-based RIs and pubertal development, median hormone by Tanner stage for key age groups are presented in Supplementary Tables S3-S6. As expected, hormone concentrations (notably LH, FSH, E2 in girls and T in boys) showed a clear progression with advancing Tanner stage within corresponding age ranges (e.g. ages 8–10 for girls, ages 10–12 for boys). This demonstrates that the significant inflection points identified in the age-specific RIs (Table 1, Figure 1) effectively capture the biochemical changes associated with pubertal maturation.

In addition, to provide a more detailed view of the hormone distributions across sexual maturity stages, descriptive statistics for all six reproductive hormones are provided in Supplementary Tables S3-S6. These tables stratify data by breast stage (B1-B5) and pubic hair stage (PH1-PH5) in females, and by genital stage (G1-G5) and pubic hair stage (PH1-PH5) in males. For Tanner stages with a sample size (n) ≥ 30, the 5th and 95th percentiles are reported. For stages with *n* < 30 (primarily stages IV and V), the observed minimum and maximum values are shown due to limited statistical robustness for percentile estimation. These data illustrate the trends of hormone changes with pubertal progression. It is important to note that due to limited sample sizes in many Tanner stage subgroups, the values in Supplementary Tables S3-S6 are presented for descriptive purposes and should not be interpreted as standalone reference intervals.

### Validation of established RIs

The verification process was performed, and all partitions successfully passed the initial verification, with no more than 2 of the 20 results falling outside the established RI for any partition. Table 2 presents the RI validation results. A summary comparing the approximate ages at which notable increases in key hormone were observed in this cohort versus a North American (CALIPER) cohort is provided in Table 3.

**Table 2.** Verification of established paediatric reference intervals for 6 reproductive hormones.

Analytes	Gender	Partition	Total samples	N, inside RI	% inside RI
E2	Male	1–3 years	20	20	100%
		4–9 years	60	60	100%
		10–13 years	39	38	97%
		14 years	20	20	100%
	Female	1–8 years	80	79	99%
		9–11 years	40	38	95%
		12–14 years	20	19	95%
T	Male	1–10 years	80	79	99%
		11 years	20	19	95%
		12–13 years	20	20	100%
		14 years	20	20	100%
	Female	1–10 years	100	100	100%
		11–14 years	40	39	98%
LH	Male	1–3 years	20	20	100%
		4–8 years	40	40	100%
		9 years	20	19	95%
		10–12 years	30	27	90%
		13–14 years	30	29	97%
	Female	1–6 years	60	59	98%
		7–8 years	20	20	100%
		9 years	20	20	100%
		10–12 years	20	19	95%
		13–14 years	20	19	95%
FSH	Male	1–6 years	40	38	95%
		7–8 years	20	18	90%
		9–10 years	20	18	90%
		11 years	20	18	90%
		12–14 years	39	37	95%
	Female	1–2 years	20	19	95%
		3–4 years	20	20	100%
		5–7 years	36	35	97%
		8–9 years	24	22	92%
		10–11 years	20	20	100%
		12–14 years	20	18	90%
PRGE	Male	1–12 years	110	108	98%
		13–14 years	29	27	93%
	Female	1–10 years	100	98	98%
		11–12 years	20	19	95%
		13–14 years	20	20	100%
PRL	Male	1–3 years	20	20	100%
		4–9 years	60	54	90%
		10–14 years	60	59	98%
	Female	1–3 years	31	31	100%
		4–9 years	69	69	100%
		10–12 years	20	19	95%
		13–14 years	20	20	100%

LH, luteinizing hormone; T, testosterone; E2, oestradiol, FSH, follicle-stimulating hormone; PRL, prolactin; PRGE, progesterone.

**Table 3.** The ages of reproductive hormones start climbing among PRINCE and CALIPER cohorts.

Analytes	Hormones start rising (Female)		Analytes	Hormones start rising (Male)	
	PRINCE (y)	CALIPER (y)		PRINCE (y)	CALIPER (y)
FSH	8	9	FSH	7	9
LH	9	11	LH	9	11
E2	9	11	T	11	12
PRGE	11	12			

PRINCE: Paediatric reference intervals in China; CALIPER: Canadian laboratory initiative on paediatric reference intervals; LH: luteinizing hormone; T: testosterone; E2: oestradiol; FSH: follicle-stimulating hormone; PRGE: progesterone.

### Correlation analysis of key hormone axes

**Figure 2.**
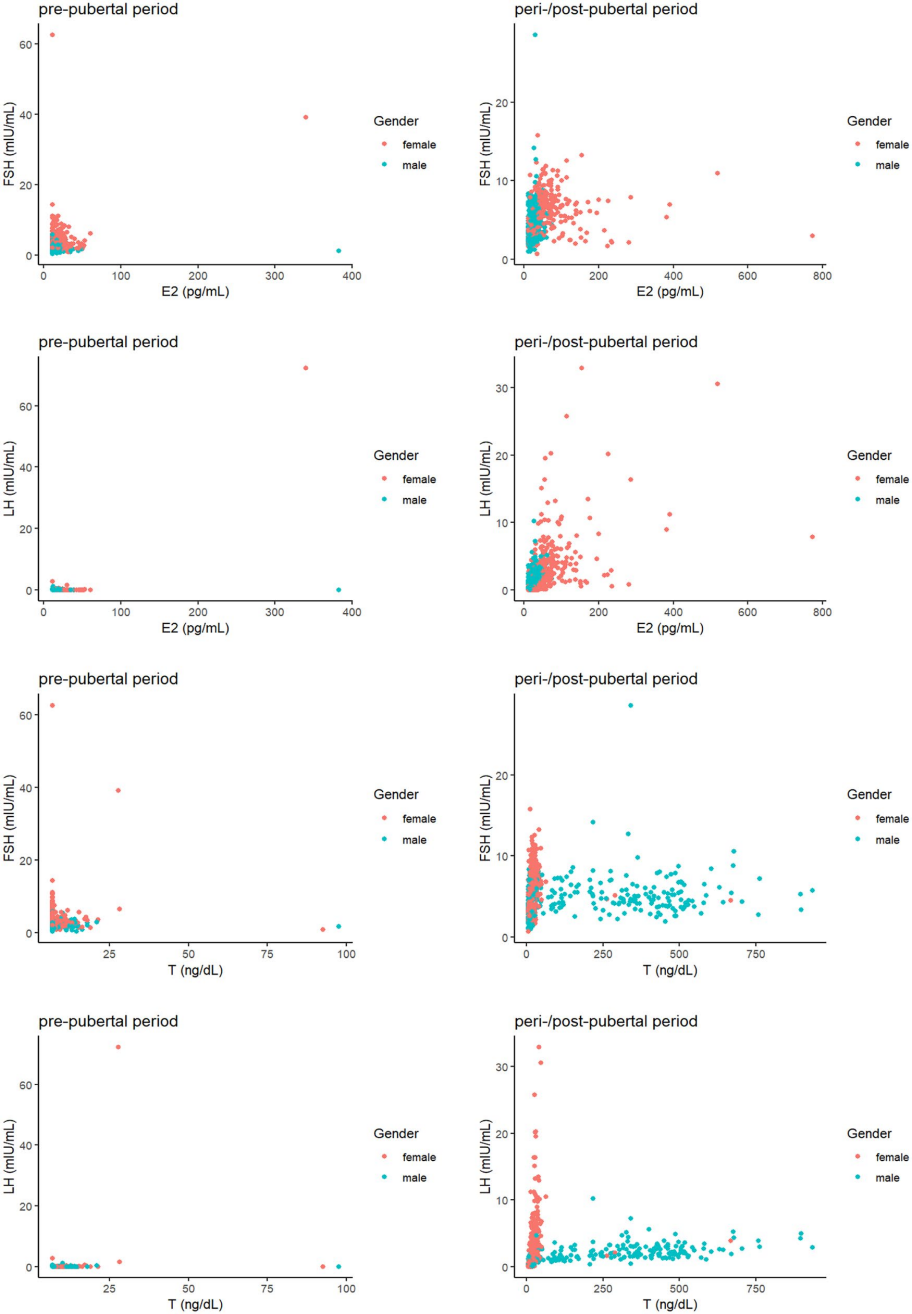
Scatter plots and correlation analyses of gonadal steroids and gonadotropins in Chinese children before and after the typical age of pubertal onset.

The correlation analyses revealed distinct patterns before and after the typical age of pubertal onset. Figure 2 illustrates these relationships. In the pre-pubertal period (0–8 years), correlations between E2 and gonadotropins (FSH, LH) and between T and gonadotropins were weak (*p* > 0.05). In contrast, during the peri-/post-pubertal period (9–14 years), significant positive correlations emerged.

## Discussion

This study established paediatric RIs for six reproductive hormones in Chinese children aged 0–14 years. The complex, age- and sex-dependent distribution of these hormones—reflecting the progressive activation of the hypothalamic-pituitary-gonadal (HPG) axis from infancy through adolescence—highlights the critical importance of stratified reference intervals when evaluating paediatric endocrine disorders.

Comparison with the North American CALIPER study [4]—which used the same analytical platform (Siemens Atellica^®^ IM)—revealed similar overall hormonal trends but notable differences in the timing of pubertal activation. Key hormones appeared to rise earlier in our Chinese cohort: LH began increasing at age 9 years in both sexes (vs. ages 11–14 in CALIPER), and increases in E2, PRGE (girls) and T (boys) occurred at younger ages (Table 3). However, this study did not measure adrenal-specific markers such as androstenedione or dehydroepiandrosterone sulphate (DHEA-S), which are more directly indicative of adrenarche. The absence of these measurements limits our ability to specifically characterize the adrenal contribution to the observed PRGE patterns. For PRL, we observed a rise in the upper limit at age 10 in girls, necessitating sex-specific partitions, whereas CALIPER presented a combined-sex RI for ages 1–19 years. These discrepancies highlight the influence of population-specific factors on HPG axis maturation and underscore the critical importance of establishing and applying population-specific RIs, even when analytical methods are aligned. The shared analytical platform strengthens the validity of these cross-population comparisons.

The age- and sex-stratified RIs for LH, FSH, E2 and T reflect progressive HPG axis activation from the suppressed pre-pubertal state to the re-awakened pubertal state [20]. The correlation analyses provide empirical support for this transition: weak correlations between gonadal steroids and gonadotropins in children aged 0–8 years shift to strong positive correlations after age 9 years, visually capturing the establishment of positive feedback loops during puberty [21] (Figure 2).

Clinical interpretation of these RIs requires consideration of several factors. First, for post-menarchal girls, the E2, LH, FSH and PRGE RIs are ‘all-phase’ intervals because samples were not timed to menstrual cycle phase. Therefore, a single elevated value may represent a normal peri-ovulatory or luteal phase measurement rather than pathology; conversely, these RIs are robust for identifying truly low levels indicative of hypogonadism. Second, as shown in Supplementary Table S2, pubertal timing varies considerably within chronological age groups. For example, only 22% of 9-year-old girls in our cohort had E2 concentrations above the pre-pubertal threshold, despite the overall surge in the upper RI limit at this age. Thus, a value above the pre-pubertal range but below the age-specific 97.5th percentile may be normal for a child who has entered puberty early within that age band. The Tanner stage-stratified data (Supplementary Tables S3-S6) provide additional context for integrating laboratory results with physical examination findings.

Our findings also support several established physiological phenomena: elevated FSH in girls before age 2 (consistent with mini-puberty), rising testosterone after age 11 in boys (driving secondary sexual development) and increasing PRL in pubertal girls (highlighting the value of sex-specific RIs for identifying hyperprolactinaemia [22]).

The observed earlier biochemical pubertal activation in our Chinese cohort compared to the North American CALIPER cohort may reflect a combination of population-specific factors. Genetic background, dietary patterns, body composition and environmental exposures can all influence the tempo of HPG axis maturation [23,24]. Additionally, differences in cohort recruitment, health screening criteria and statistical partitioning methods may contribute to the apparent age differences in hormonal surges. Therefore, while our data suggest an earlier transition in this Chinese population, this finding should not be overgeneralized; it likely represents a composite of true physiological variation, secular trends and methodological nuances. Most importantly, these observations reinforce our primary conclusion: population-specific RIs calibrated with local methods are essential for accurate clinical assessment.

The age- and sex-specific RIs established here provide an evidence-based tool for paediatric endocrinology practice in China. Their primary clinical utility lies in objective interpretation of laboratory results in the context of growth and development. Key principles for clinical application include: (1) RIs are most powerful when combined with auxology and Tanner staging. For example, a 10-year-old girl with Tanner stage B3 breast development can have her oestradiol level compared against both the age-specific RI and the typical range for Tanner stage B3 (Supplementary Tables S3-S6). Discordance between these references warrants further investigation; (2) As shown in Supplementary Table S2, even within age groups where upper RI limits surge (e.g. E2 at age 9 in girls), only a subset of individuals have entered puberty biochemically (22% in our cohort). Therefore, a value above the pre-pubertal range but below the age-specific 97.5th percentile may be normal for a child who has entered puberty early within that age band; (3) The ‘all-phase’ RIs for E2, LH, FSH and PRGE are robust for identifying hypogonadism but cannot distinguish cycle phase-specific abnormalities. A single elevated value may represent normal peri-ovulatory or luteal phase physiology; and (4) These RIs can serve as a baseline for monitoring children receiving therapies affecting the HPG axis (e.g. GnRH analogues for central precocious puberty), with the expected trajectory being a return to the pre-pubertal range for chronological age. In summary, these RIs provide a population-based framework that, when combined with clinical assessment, enables nuanced evaluation of endocrine status and guides decisions on observation, further investigation, or intervention.

Several limitations of this study should be acknowledged. First, the sample size, while adequate for RI establishment per CLSI guidelines, was modest for certain subgroups. Larger multi-center studies would enable more granular age partitions and improve generalizability. Second, the verification of the established RIs, while performed according to CLSI guidelines, utilized a modest sample size of 20 per partition. This process is best interpreted as a successful check for gross transferability rather than a high-precision re-validation, as verification with 20 samples per partition provides limited statistical power. Future multi-center studies with larger verification cohorts would further strengthen confidence in these intervals. Third, blood sampling was not standardized to menstrual cycle phase in post-menarchal girls. While this reflects real-world clinical practice, the resulting ‘all-phase’ RIs for E2, LH, FSH and PRGE have increased biological variance and are robust for identifying profoundly abnormal values but may have reduced sensitivity for detecting phase-specific disturbances. Fourth, the cross-sectional design with limited sampling in the first months of life precluded detailed characterization of mini-puberty; the RIs for the youngest age group provide a useful baseline but are not intended for diagnosing disorders related to this transient phase. Fifth, the immunoassays used, while meeting quality standards, may have limitations at very low concentrations (particularly for E2 in pre-pubertal children), as evidenced by sporadic implausibly high values that were treated as outliers. Mass spectrometry-based methods may offer improved specificity for determining lower limits of normal in pre-pubertal populations. Finally, we did not measure adrenal markers (e.g. DHEA-S), limiting our ability to characterize the adrenal contribution to observed PRGE patterns. Finally, for the small number of samples identified as statistical outliers with physiologically implausibly high hormone concentrations, confirmatory testing using mass spectrometry-based methods was not performed due to sample volume limitations and resource constraints. Such methods may offer improved specificity for resolving true elevated concentrations from assay interference or cross-reactivity, particularly at the extreme ends of the measuring range. Future studies incorporating LC/MS/MS confirmation for outlier values would strengthen the validity of the reference intervals.

## Conclusion

This study used data from the PRINCE project to establish, for the first time, RIs for reproductive hormones in Chinese children. However, we recommend conducting a small-sample validation per the industry standards (WS/T402) before using these RIs in clinical practice. These findings provide evidence-based data for interpreting laboratory test results and establishing clinical diagnostic criteria for paediatric endocrine diseases. Finally, comparisons with a North American cohort underscore the importance of population-specific RIs.

## Supplementary Material

Supplemental Material

Figure S1.tif

## Data Availability

The datasets used and/or analysed for the current study are available from the corresponding author on reasonable request.
