# Supplementary material for: Reference intervals for reproductive hormones in Chinese children aged 0–14 years based on the PRINCE study
Source: Ann Med. 2026 Apr 10;58(1):2650008. doi: 10.1080/07853890.2026.2650008 (PMC13072677; doi:10.1080/07853890.2026.2650008)
Supplement: Supplemental Material [file IANN_A_2650008_SM8314.docx]

Table S1. Performance parameters of the chemiluminescence analyzer.

|  | LH | FSH | T | E2 | PRGE | PRL |
| --- | --- | --- | --- | --- | --- | --- |
|  | mIU/mL | mIU/mL | ng/dL | pg/mL | ng/mL | ng/mL |
| Limit of Blank | 0.07 | 0.2 | 1.81 | 7.33 | 0.18 | 0.06 |
| Limit of Detection | 1.44 | 0.55 | 4.43 | 11.54 | 0.31 | 0.35 |
| Limit of Quantitation | / | / | 4.43 | / | / | / |
| Measuring interval | 0.07-200 | 0.30-200 | 7.00-1500 | 11.80-3000 | 0.21-60 | 0.30-200 |
| Coefficient of variation within the measuring interval | 2.1-3.4% | 2.6-8.2% | 2.7-6.7% | 2.7-14.7% | 2.9-12.9% | 2.3-4.9% |

Table S2. Proportion of Individuals with Hormone Concentrations Above a Pre-pubertal Threshold in Key Age Groups

| Gender | Analytes | Partition | Pre-pubertal Upper Limits | N | ≥pre-pubertal upper limit | |
| --- | --- | --- | --- | --- | --- | --- |
|  |  |  |  |  | n | ratio |
| Female | E2 (pg/mL) | 9 years | 39.5 | 92 | 20 | 22% |
|  | LH (mIU/mL) | 9 years | 0.23 | 92 | 27 | 29% |
| Male | LH (mIU/mL) | 9 years | 0.25 | 78 | 15 | 19% |
|  | T (ng/dL) | 11years | 18.59 | 40 | 20 | 50% |

Table S3: Female Breast Tanner I-V for reproductive hormones

| Breast | n | Ratio | FSH (mIU/mL) | | LH (mIU/mL) | | PRGE (ng/mL) | | PRL(ng/mL) | | T(ng/dL) | | E2 (pg/mL) | |
| --- | --- | --- | --- | --- | --- | --- | --- | --- | --- | --- | --- | --- | --- | --- |
|  |  |  | 5% | 95% | 5% | 95% | 5% | 95% | 5% | 95% | 5% | 95% | 5% | 95% |
| TannerⅠ | 106 | 12% | 1.72 | 6.86 | 0.00 | 1.06 | 0.09 | 0.51 | 2.96 | 13.28 | 7.00 | 18.83 | 11.80 | 51.23 |
| Tanner Ⅱ | 92 | 10% | 1.60 | 9.65 | 1.60 | 6.19 | 0.09 | 0.77 | 3.19 | 14.19 | 7.00 | 31.66 | 11.80 | 99.16 |
| Tanner Ⅲ | 85 | 10% | 1.99 | 10.32 | 0.48 | 14.66 | 0.15 | 11.26 | 3.78 | 25.07 | 12.77 | 39.42 | 21.76 | 224.70 |
| Tanner Ⅳ* | 21 | 2% | 1.98 | 13.21 | 0.00 | 32.95 | 0.20 | 4.73 | 5.30 | 19.39 | 11.51 | 48.65 | 29.72 | 772.51 |
| Tanner Ⅴ* | 3 | 0% | 6.77 | 8.32 | 2.05 | 4.81 | 0.19 | 1.68 | 7.99 | 8.92 | 19.65 | 24.94 | 60.93 | 77.23 |
| No information | 580 | 65% |  |  |  |  |  |  |  |  |  |  |  |  |
| 1-7 years | 453 | 51% |  |  |  |  |  |  |  |  |  |  |  |  |
| 8-14 years | 127 | 14% |  |  |  |  |  |  |  |  |  |  |  |  |
| Total | 887 | 100% |  |  |  |  |  |  |  |  |  |  |  |  |

Table S4: Female Pubic hair Tanner I-V for reproductive hormones

| Pubic hair | n | Ratio | FSH (mIU/mL) | | LH (mIU/mL) | | PRGE (ng/mL) | | PRL(ng/mL) | | T(ng/dL) | | E2 (pg/mL) | |
| --- | --- | --- | --- | --- | --- | --- | --- | --- | --- | --- | --- | --- | --- | --- |
|  |  |  | 5% | 95% | 5% | 95% | 5% | 95% | 5% | 95% | 5% | 95% | 5% | 95% |
| TannerⅠ | 164 | 18% | 1.67 | 8.55 | 0.00 | 3.64 | 0.09 | 0.52 | 3.03 | 13.65 | 7.00 | 25.63 | 11.80 | 70.93 |
| Tanner Ⅱ | 75 | 8% | 1.77 | 11.22 | 0.00 | 14.02 | 0.11 | 7.95 | 3.53 | 16.37 | 7.15 | 37.21 | 11.80 | 200.62 |
| Tanner Ⅲ | 54 | 6% | 2.37 | 10.74 | 0.74 | 19.89 | 0.16 | 10.45 | 4.71 | 24.94 | 13.80 | 44.50 | 25.42 | 310.44 |
| Tanner Ⅳ* | 11 | 1% | 1.98 | 10.85 | 0.00 | 12.92 | 0.19 | 17.17 | 5.30 | 33.21 | 11.51 | 48.65 | 29.72 | 235.46 |
| Tanner Ⅴ* | 1 | 0% | 8.32 | 8.32 | 4.48 | 4.48 | 1.68 | 1.68 | 8.41 | 8.41 | 21.87 | 21.87 | 60.93 | 60.93 |
| No information | 582 | 66% |  |  |  |  |  |  |  |  |  |  |  |  |
| 1-7 years | 453 | 51% |  |  |  |  |  |  |  |  |  |  |  |  |
| 8-14 years | 129 | 15% |  |  |  |  |  |  |  |  |  |  |  |  |
| Total | 887 | 100% |  |  |  |  |  |  |  |  |  |  |  |  |

Table S5: Male Pubic hair Tanner I-V for reproductive hormones

| Pubic hair | n | Ratio | FSH (mIU/mL) | | LH (mIU/mL) | | PRGE (ng/mL) | | PRL(ng/mL) | | T(ng/dL) | | E2 (pg/mL) | |
| --- | --- | --- | --- | --- | --- | --- | --- | --- | --- | --- | --- | --- | --- | --- |
|  |  |  | 5% | 95% | 5% | 95% | 5% | 95% | 5% | 95% | 5% | 95% | 5% | 95% |
| TannerⅠ | 112 | 14% | 1.48 | 7.20 | 0.00 | 2.49 | 0.12 | 0.45 | 2.92 | 18.12 | 7.00 | 412.17 | 11.80 | 30.20 |
| Tanner Ⅱ | 85 | 10% | 1.56 | 8.49 | 0.00 | 3.38 | 0.04 | 0.68 | 3.11 | 13.31 | 7.00 | 492.85 | 11.80 | 43.16 |
| Tanner Ⅲ | 33 | 4% | 1.92 | 9.07 | 0.11 | 4.97 | 0.12 | 1.00 | 17.89 | 17.89 | 7.00 | 802.23 | 11.80 | 65.44 |
| Tanner Ⅳ* | 13 | 2% | 2.86 | 12.68 | 0.08 | 4.44 | 0.12 | 0.88 | 3.20 | 15.34 | 8.20 | 704.86 | 11.80 | 49.83 |
| Tanner Ⅴ* | 4 | 0% | 3.37 | 5.60 | 0.86 | 4.95 | 0.39 | 0.74 | 5.44 | 19.66 | 120.86 | 896.85 | 16.85 | 45.25 |
| No information | 572 | 70% |  |  |  |  |  |  |  |  |  |  |  |  |
| 1-7 years | 437 | 53% |  |  |  |  |  |  |  |  |  |  |  |  |
| 8-14 years | 135 | 16% |  |  |  |  |  |  |  |  |  |  |  |  |
| Total | 819 | 100% |  |  |  |  |  |  |  |  |  |  |  |  |

Table S6: Male Pubic hair Tanner I-V for reproductive hormones

| Testis | n | Ratio | FSH (mIU/mL) | | LH (mIU/mL) | | PRGE (ng/mL) | | PRL(ng/mL) | | T(ng/dL) | | E2 (pg/mL) | |
| --- | --- | --- | --- | --- | --- | --- | --- | --- | --- | --- | --- | --- | --- | --- |
|  |  |  | 5% | 95% | 5% | 95% | 5% | 95% | 5% | 95% | 5% | 95% | 5% | 95% |
| Tanner G1 | 114 | 14% | 1.44 | 7.08 | 0.00 | 3.15 | 0.12 | 0.53 | 3.06 | 16.35 | 7.00 | 471.59 | 11.80 | 35.91 |
| Tanner G2 | 89 | 11% | 1.71 | 7.69 | 0.00 | 3.84 | 0.04 | 0.89 | 3.01 | 14.03 | 7.00 | 656.07 | 11.80 | 47.67 |
| Tanner G3 | 34 | 4% | 1.46 | 9.61 | 0.00 | 4.79 | 0.12 | 0.98 | 25.97 | 25.97 | 7.00 | 677.37 | 11.80 | 55.59 |
| Tanner G4* | 12 | 1% | 2.96 | 6.81 | 0.05 | 4.90 | 0.14 | 0.74 | 13.99 | 704.86 | 13.99 | 704.86 | 12.54 | 44.47 |
| Tanner G5* | 1 | 0% | 2.71 | 2.71 | 2.65 | 2.65 | 0.29 | 0.29 | 438.15 | 438.15 | 438.15 | 438.15 | 31.86 | 31.86 |
| No information | 569 | 69% |  |  |  |  |  |  |  |  |  |  |  |  |
| 1-7 years | 437 | 53% |  |  |  |  |  |  |  |  |  |  |  |  |
| 8-14 years | 132 | 16% |  |  |  |  |  |  |  |  |  |  |  |  |
| Total | 819 | 100% |  |  |  |  |  |  |  |  |  |  |  |  |

Supplementary Figure S1: Population-derived smoothed percentile curves for reproductive hormones in Chinese children. Smoothed curves representing the central tendency (median or geometric mean) and the 2.5th and 97.5th percentiles for Luteinizing Hormone, Follicle-Stimulating Hormone (FSH), Estradiol (E2), and Testosterone (T) are shown for the study cohort. These trajectories provide context for the individual trends observed in the main figures by illustrating the normative distribution of hormone levels across the age range.
