# Supplementary figures and images for: Reference intervals for reproductive hormones in Chinese children aged 0–14 years based on the PRINCE study
Source: Ann Med. 2026 Apr 10;58(1):2650008. doi: 10.1080/07853890.2026.2650008 (PMC13072677; doi:10.1080/07853890.2026.2650008)

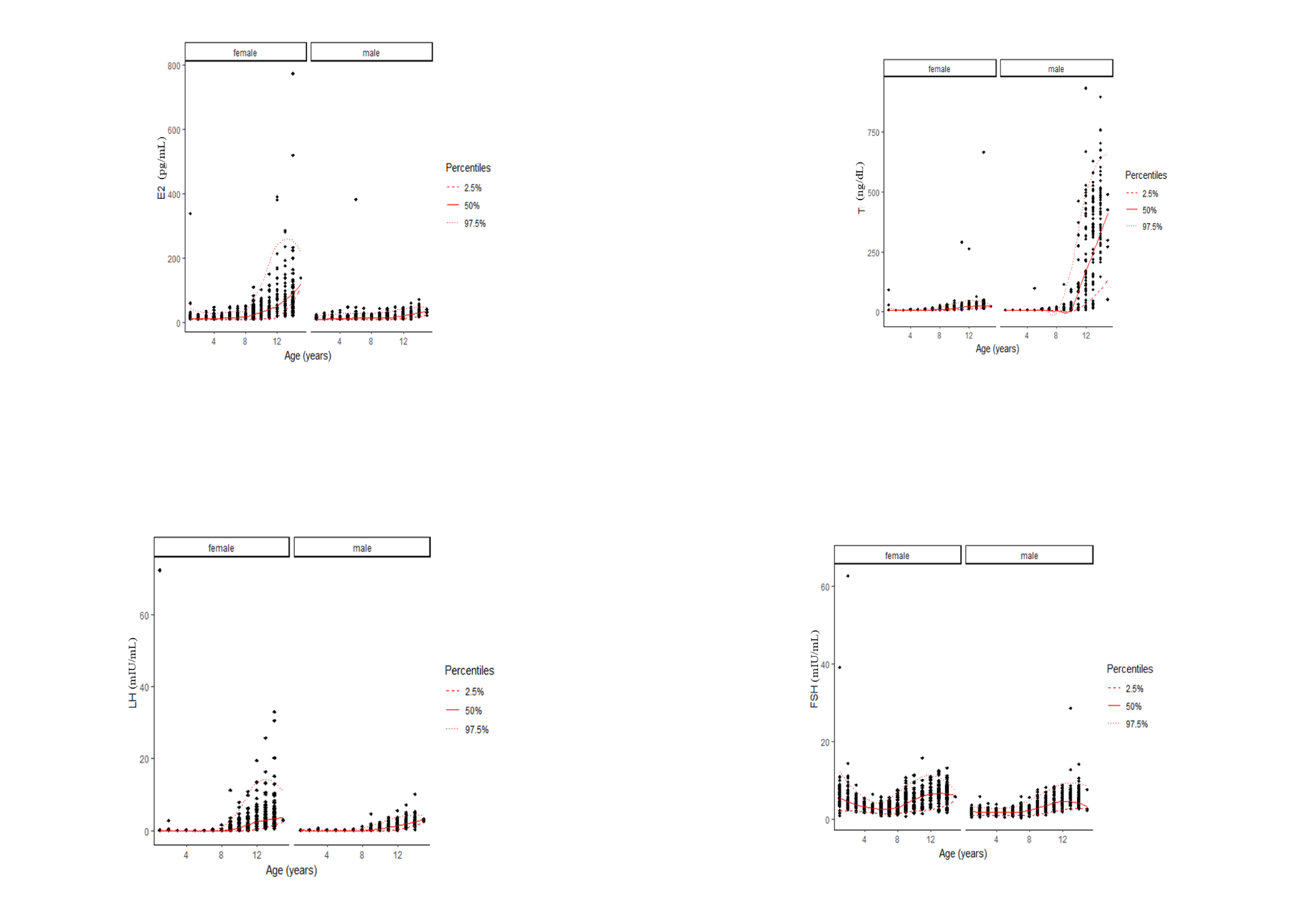

Supplement: Figure S1.tif [file IANN_A_2650008_SM7897.tif]
